# Simple Ion–Gas Mixtures as a Source of Key Molecules Relevant to Prebiotic Chemistry

**DOI:** 10.3390/molecules26237394

**Published:** 2021-12-06

**Authors:** Samuel Paula, Liam S. Goulding, Katherine N. Robertson, Jason A. C. Clyburne

**Affiliations:** Department of Chemistry, Saint Mary’s University, Halifax, NS B3H 3C3, Canada; samuelpaula2121@gmail.com (S.P.); liamgoulding95@gmail.com (L.S.G.); katherine.robertson@smu.ca (K.N.R.)

**Keywords:** prebiotic, origins of life, wet dry cycle, interface, membrane, inorganic chemistry, volatile sulfur compounds, main group chemistry, radical anion, EPR, hydrothermal systems, small molecule activation, abiogenesis

## Abstract

Very simple chemistry can result in the rapid and high-yield production of key prebiotic inorganic molecules. The two reactions investigated here involve such simple systems, (a) carbon disulfide (CS_2_) and acetate (CH_3_COO¯) and (b) sulfur dioxide (SO_2_) and formate (HCOO¯). They have been carried out under non-aqueous conditions, either in an organic solvent or with a powdered salt exposed to the requisite gas. Under such dry conditions the first reaction generated the thioacetate anion [CH_3_COS]¯ while the second produced the radical [SO_2_^·^]¯anion. Anhydrous conditions are not rare and may have arisen on the early earth at sites where an interface between different phases (liquid/gas or solid/gas) could be generated. This is one way to rationalize the formation of molecules and ions (such as we have produced) necessary in the prebiotic world. Interpretation of our results provides insight into scenarios consistent with the more prominent theories of abiogenesis.

## 1. Introduction

Renewed interest in prebiotic chemistry and the chemical origins of life has raised many seemingly unanswerable questions and produced a variety of seemingly plausible, but possibly unprovable, explanations [1,2,3]. Typically, the area of prebiotic chemistry has been the purview of organic chemists, but simple inorganic reactions are likely to also have played key roles. Unfortunately, there is no *molecular* fossil record to document the transition from small *inorganic* molecules to larger *organic* molecules. Recent discoveries suggest that this shift could have occurred at warm hydrothermal vents, such as are found at the Lost City system [4,5,6,7,8,9]. This environment presents an opportunity for the simplest phases of matter, including salts, molecular solids, and gases to commingle and possibly react [10]. The warm outflow from such vents (T < 40 °C to 90 °C) is methane- and hydrogen-rich and is markedly alkaline (pH 9 to 11). Through processes including serpentinization [11,12] the water contains a variety of reactive small molecules, gases and salts, conditions which may have promoted the initiation of relevant chemical transformations. Alternatively, fluctuating volcanic hot spring pools may have been the site for the origin of many of the molecules of life, since the cyclic process of pool formation and drying provides an opportunity for concentration, reaction, and possibly replication. In other theories, aerosol formation has been invoked as a source of the conditions necessary for important reactions to have been initiated [13]. All of these scenarios suggest that for key reactions to have occurred, there must have been dry conditions where phase separated reactants could combine.

How could phase separation(s) have been established involving the ionic and gaseous components meeting and what would the consequences have been if this had occurred? A possible answer to this was recently published, describing how gas bubbles in water subjected to a thermal gradient drive enrichment of prebiotic molecules at hydrothermal vents [14]. Sleep et al. have predicted that serpentinization will produce hydrogen-rich water from which hydrogen bubbles can form [15]. They have also predicted that when this hydrogen-rich water mixes with the CO_2_-rich vent fluids, methane gas should be produced. The pores present in the columnar vents offer a site where anhydrous chemistry could occur once a phase separation (gas bubble formation) had been established [16]. We also note here that there is a pronounced phase separation in the dispersed solid–gas interface observed for aerosols, and some of our results will be relevant to this broad area [17,18]. Dispersion of the salty aerosol, coupled with evaporation, will generate an anhydrous environment for gas-ion chemistry (in an oxygen free environment), and one such reaction is reported in this work.

Our interest in this area comes not from context of the organic chemical origin of life, but rather from the inorganic perspective. Central to many speculated prebiotic reactions are small inorganic molecules, all of which must have been found in close proximity to the organic species for reactions to have occurred. General interest in these inorganics resurged when it was reported that Venus has low atmospheric concentrations of phosphine PH_3_, generated geologically, chemically, or biologically [19]. Doubt has been cast on the veracity of the detection [20], but it does make clear that much of the chemistry of these key small molecules remains to be explored.

Many inorganic small molecules have extremely short lifetimes under aqueous conditions. This has led to one of the most stubborn problems facing prebiotic scientists, the so-called “water problem” as discussed by Benner et al. in relation to the synthesis of RNA but applicable to many facets of chemical evolution [21]. The problem is diabolically simple, even for organic molecules; many bonds are thermodynamically unstable with respect to hydrolysis in water. Thus, even if these are made in water, they fall apart. Our experience with a wide variety of organic and organometallic reagents in ionic media has shown that the chemistry of dry ionic materials is drastically different from that which occurs in either aqueous solutions or in wet ionic phases. This led us to consider whether an anhydrous environment might increase the potential for reactivity (other than hydrolysis) for simple gases and ions and whether conversion of these small species to larger, more biologically relevant materials might then be possible.

The fact that the gases and molecules in the prebiotic world would have substantially different (anhydrous) chemistries compared to their better-known aqueous chemistry appears to have been overlooked. Furthermore, the chemistry proposed in any theory must match the conditions under which it is expected to occur. For instance, carbonyl sulfide (COS) [22] has been invoked as a potential coupling reagent for amino acids to produce peptides under prebiotic conditions [23]. However, COS has a very short lifetime in basic aqueous media; its non-polar cousin, carbon disulfide (CS_2_), is more persistent [24]. In addition, the utility of COS for peptide-coupling under aqueous conditions is concomitantly lowered since a significant fraction will be hydrolyzed under basic conditions. Lastly, elemental oxygen was not available in high concentrations at these prebiotic times, making conversion of appreciable quantities of CS_2_ to COS unlikely.

Phase separation is a concept well appreciated in both industrial and laboratory settings, but phase separation is also central for much of the chemistry found in nature. For instance, many enzyme reactions occur in an aqueous environment, but it is an important distinction that most of the chemistry occurring at the active sites of biological molecules is markedly anhydrous [25]. In many enzymes water is intimately involved with chemical activity, but the water molecules are delivered in a controlled fashion. The reactivity observed is not that of bulk water but more closely aligned to the molecular addition of single water molecules. The low dielectric constant of enzymatic sites broadly parallels that of anhydrous organic/organometallic solids and ionic liquids and one can speculate that perhaps this environment is a trace or fossil of primitive chemistry/biology. Active sites of enzymes have obviously been selected through evolutionary processes; however, it does suggest that this type of hydrophobic environment may also have been present where primitive chemistry was occurring, vis-à-vis the hydrothermal vents, volcanic fields, etc. In addition, small molecules, such as H_2_S, COS, NO, NH_3_, CH_4_, and SO_2_, are still important signaling molecules for all known life forms [26] and their chemistry continues to provide surprises [27].

**Scheme 1:**
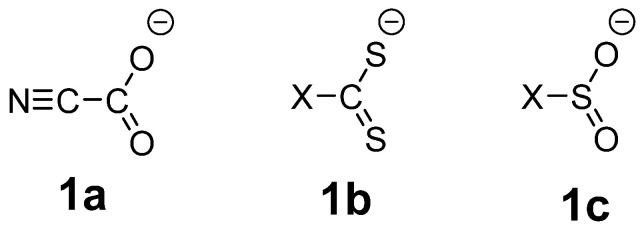
Anions relevant to this paper.

We have spent some time exploring the chemistry of small anions with structurally simple gasses. For example, we identified the cyanide complex of CO_2_, a species that is extremely fragile but is implicated in the ripening processes of fruit [25]. Little is known of the reactions between carbon dioxide and halides or pseudohalides (e.g., OH^−^, CN^−^, OCN^−^, SCN^−^, and N_3_^−^), although this lack of knowledge can be attributed, at least in part, to the instability of these donor–acceptor complexes [28]. Likewise, many other complexes expected to be formed between simple organic anions and small gases (Scheme 1) have never been isolated. In this work we report very simple reactions that occur between gas molecules and small, univalent anions. These reactions are so simple, in fact, that one would have presupposed that they had been characterized and would have no further secrets to reveal. Quite the opposite has proven to be true: our observations have led us to wonder what other simple chemistry may have similarly been overlooked. Independent of the model chosen (hydrothermal vent, volcanic pond, aerosol), anhydrous ion/gas chemistry has not before been considered as a possible contributor to the evolution of prebiotic compounds.

## 2. Results and Discussion

The specific reactions we have investigated involve the combinations of (1) carbon disulfide gas (CS_2_) and the acetate anion (CH_3_COO¯) and (2) sulfur dioxide gas (SO_2_) and the formate anion (HCOO¯). These reactions were carried out under non-aqueous conditions, either in organic solvent(s) or with a solid salt exposed to the required gas. The chemistry of these two systems is related to some well-described processes (see references herein); what has not previously been appreciated is how the products from these simple ion-rich reactions might be considered in the context of prebiotic chemistry.

**Scheme 2:**
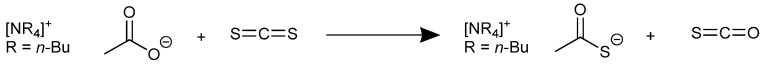
The reaction between solid tetrabutylammonium acetate and CS_2_ gas.

We began by studying the reaction between solid tetrabutylammonium acetate, ([*n*-Bu_4_N][CH_3_COO]), and CS_2_ gas (Scheme 2). The adduct expected to form, [CH_3_COOCS_2_], was not one of the observed products. However, the powdered solid did react rapidly with CS_2_ vapor in a reaction that could have formed the CS_2_ adduct as an undetected intermediate. The reaction mixture turned red initially and then after several hours turned yellow. The resulting sticky material could be recrystallized in modest yield. Infrared studies of the atmosphere over the solid showed that COS gas was being formed, as indicated by characteristic adsorption bands observed at 2071 and 2053 cm^−1^ (Figure S1). These peaks appeared upon the addition of CS_2_ and grew in intensity as the reaction progressed.

**Figure 1:**
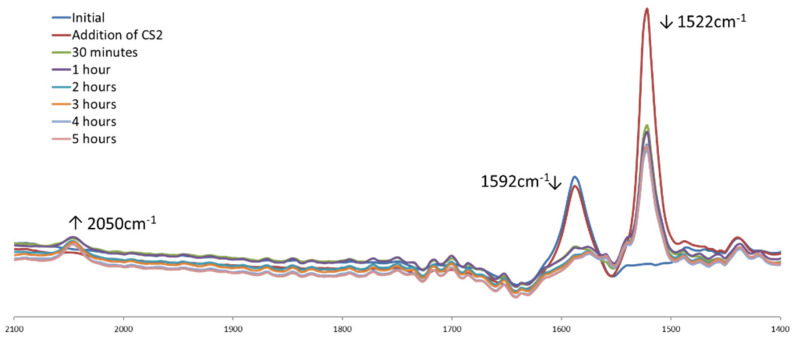
ReactIR monitoring of the reaction between tetrabutylammonium acetate and CS_2_ in acetonitrile solution. The changes in the IR spectra over time show the loss of acetate (1592 cm^−1^) and CS_2_ (1522 cm^−1^) along with the production of COS (2050 cm^−1^).

**Figure 2:**
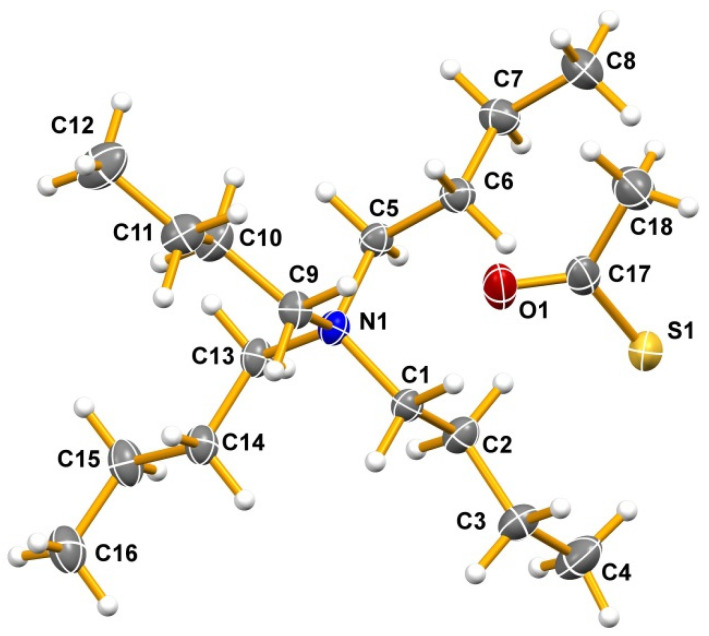
The crystal structure of tetrabutylammonium thioacetate. Thermal ellipsoids have been drawn at the 50% probability level and hydrogen atoms have not been labelled. Selected bond lengths (Å) and angles (°): S(1)-C(17) = 1.7225(15), O(1)-C(17) = 1.2358(15), C(18)-C(17) = 1.517; S(1)-C(17)-O(1) = 124.96(11), S(1)-C(17)-C(18) = 117.27(10), O(1)-C(17)-C(18) = 117.76(13).

This reaction also proceeded smoothly in acetonitrile, with the same characteristic color changes. In situ infrared monitoring (ReactIR) was used to observe the generation of COS in the acetonitrile solution (Figure 1). Two peaks were tracked, one at 1522 cm^−1^ that is indicative of CS_2_ (ν_3_ asymmetric stretch) and one at 2050 cm^−1^ that corresponds to COS (ν_3_ asymmetric stretch). We observed *quantitative* O/S exchange, generating a thioacetate salt (confirmed by X-ray crystallography and shown in Figure 2) and carbonyl sulfide gas, COS (Figure S3 of the Supplementary Materials). Infrared studies on the product from the solid-state reaction also confirmed the presence of the thioacetate ion.

The crystallographic study revealed a thioacetate anion having a geometry consistent with that of other known examples (see Tables S1 and S2 and Figures S4 to S7 of the Supplementary Materials). A discussion of the intermolecular interactions is also presented in the Supplementary Materials. To the limit of the sum of the van der Waals radii, each anion interacts with three cations, with more of these contacts involving the larger, softer sulfur atom than the smaller and harder oxygen atom of the anion. In the cation, the hydrogen atoms on carbon atoms adjacent to the central nitrogen atom are the most likely to act as hydrogen bond donors.

Initial experiments indicated that the rate of reaction in solution is proportional to the concentrations of both of the reactants. The reaction in the solid phase was likewise rapid and was finished in hours. This same reaction was not observed in water; this is understandable if the first interaction between the acid gas and the anion involves formation of a coordination complex. Protonation would significantly decrease the basicity of the acetate ion, and therefore decrease its ability to engage with the carbon disulfide. Lastly, the highly basic ionic liquid 1-butyl-3-methylimidazolium acetate has previously been shown to react with carbon disulfide, giving products consistent with those described here [29,30]. Our results show that the reaction is not limited to ionic liquids and more importantly, it can occur at the interface between a solid and the gas.

In a similar fashion, bis(triphenylphosphoranylidene)ammonium formate ([PNP]-[HCO_2_]) dissolved in acetonitrile was reacted with sulfur dioxide gas. A rapid exothermic reaction with concomitant evolution of gaseous products was observed. When the same [PNP][HCO_2_] salt was exposed to sulfur dioxide in a dry environment, a similar immediate exothermic reaction that visibly evolved gas(es) was observed (infrared spectra are shown in Figure S8 of the Supplementary Materials). The gaseous products have been identified as molecular hydrogen and carbon dioxide, using gas chromatography (Section 3.5) and infrared spectroscopy (Figure S9 of the Supplementary Materials), respectively.

**Figure 3:**
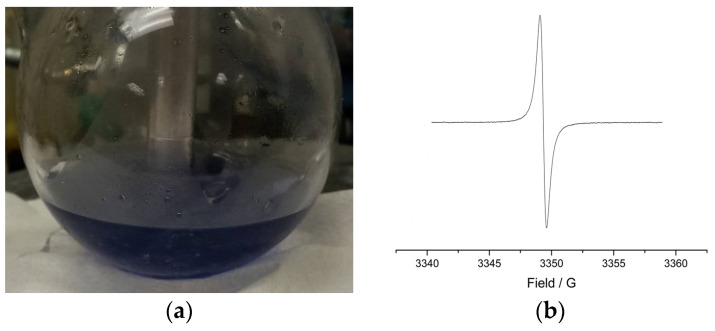
(**a**) Blue solution indicative of formation of the sulfur dioxide radical anion and (**b**) solution EPR spectrum from the reaction of sulfur dioxide, sodium formate and 18-crown-6 in acetonitrile.

Upon the addition of the sulfur dioxide gas to [PNP][HCO_2_], the reaction solution immediately turned an intense blue color (Figure 3a) then returned to clear and colorless within an hour. This blue color likely results from the formation of the sulfur dioxide radical anion, [SO_2_^‧^]¯ [31,32]. Electron paramagnetic resonance (EPR) studies were carried out on solutions of sodium formate and 18-crown-6 (2:1 ratio) in acetonitrile. Addition of SO_2_ to the sample resulted in the observation of one strong peak with a linewidth of roughly 3 G (Figure 3b). This corresponds to a calculated g value of 2.006, consistent with our speculation that this chemistry is dominated by the formation of the [SO_2_^‧^]¯ radical anion. Although the subsequent chemistry of this short-lived species is complex, it has been shown to produce polysulfate ions, including [S_2_O_4_]^2−^ and [S_4_O_6_]^2−^ [33]. The original reaction between the formate and the gas must result in a reduction of the sulfur, from a high oxidation state starting material to a lower oxidation state product. A second reaction was also studied by EPR. A solid sample of a formate salt in 90% solid NaCl gave a very weak signal, likely due to the formation of a paramagnetic species. The [SO_2_^‧^]¯ radical anion has a potentially important role in numerous areas of chemistry, noting the rapid recent advances in anodic synthetic reactions of value added and biologically active molecules [34]. We suggest that the formation of such radical species may be relevant to atmospheric chemistry as well as prebiotic chemistry.

As predicted by Russell, the discovery of warm alkaline hydrothermal vents has revolutionized our modern theories of primitive or prebiotic chemistry [35]. These vents are rich in molecules that are predisposed [36], given the appropriate conditions, to assemble many of the key components of prebiotic systems, but our results clearly show an important nuance to this model. So too does the volcanic field hypothesis merge nicely with the chemistry reported in this paper, since they both suggest the possible existence of dry anoxic environments suitable for anhydrous reactions to occur.

Phase partitioning and favorable changes in entropy could have been the driving forces required to promote much of the chemistry invoked in the prebiotic context. A compelling system which could be envisioned would involve a gas bubble in the chemically rich aqueous environment of a hydrothermal vent. The interior of the bubble would be exceptionally dry, allowing moisture sensitive reactions to proceed, although pressure would affect both the rate and the extent of reaction. Phase partitioning would occur, with hydrophobic molecules/ions moving from the aqueous environment to the bubble surface and then into the interior of the bubble. If such a bubble were to move or to form inside a pore of the hydrothermal vent, then an extra degree of complexity would be possible with the solid phase being available to interface with the gas (bubble) and/or the aqueous environment. An even greater range of anhydrous chemistry would be possible with the introduction of a gas–solid interface to the system. It would provide a unique chemical environment where chemical reactions, such as dehydrations and condensations, would be more likely to occur.

Carbon disulfide is the major product of reactions involving transition metal sulfides, with other thiols formed in smaller proportions [37,38]. Carbon disulfide is considerably more stable in water than is COS but once inside a dry, acetate containing interface it would have readily been converted to carbonyl sulfide and thioacetate. Both of these are key prebiotic reagents that are, respectively, a catalyst for amino acid coupling reactions and a component of, and possible precursor to, acetyl-CoA. The reaction of formate with sulfur dioxide provides an easy reaction pathway for the generation of the sulfur dioxide anion radical, which can then go on to produce a variety of poly-oxo-anions [39].

All of these scenarios suggest that simple reactions carried out under anhydrous and phase separated conditions could lead to a variety of transformations rich in chemical potential. The results that we have presented show that reactions carried out under anhydrous, phase separated conditions produce products quite different from those recovered from aqueous media.

## 3. Materials and Methods

All preparations were performed under an inert nitrogen atmosphere in either an MBraun glove box (M. Braun Incorporated, Stratham, NH, USA) or by standard Schlenk line techniques, unless otherwise stated. Tetrabutylammonium acetate (97%), bis(triphenylphosphoranylidene)ammonium chloride (97%), sodium chloride, acetonitrile (99.8%, anhydrous) and sodium formate (98%) were purchased from Sigma Aldrich Co. Canada (Oakville, ON, Canada) and used without further purification. Carbon disulfide was purchased from Sigma Aldrich and was dried before use with magnesium sulfate. Sulfur dioxide and nitrogen gases (>99.998%) were provided by Praxair Canada (Mississauga, ON, Canada). Reagent grade acetonitrile, dichloromethane and acetone were purchased from Caledon Laboratory Chemicals (Georgetown, ON, Canada). Magnesium sulfate and potassium bromide (infrared grade) were purchased from Fisher Scientific Canada (Ottawa, ON, Canada) and were dried/stored in an oven prior to use. Benzene-*d*_6_ (D: 99.5%), dichloromethane-*d*_2_ (D: 99.8%), acetonitrile-*d*_3_ (D: 99.6%) and DMSO-*d*_6_ (D: 99.9%) were purchased from Cambridge Isotope Laboratories, Inc. (Tewksbury, MA, USA) and were opened and stored under nitrogen.

Infrared spectra were collected using a Bruker Vertex 70 Infrared Spectrometer (Bruker Optics Ltd., Milton, ON, Canada) as either liquid films on sodium chloride salt plates, KBr pellets or within a gas IR chamber having sodium chloride salt plates as its windows. Data processing was completed using the OPUS 6.0 software suite. In situ infrared spectra were collected using a Mettler Toledo ReactIR 15 (Mettler Toledo Canada, Mississauga, ON, Canada). The data were initially analyzed using iC.IR 4.3 and plotted in Microsoft Excel 2010. Unless otherwise stated the reactions were performed under nitrogen gas and with gentle stirring throughout the course of the reaction.

NMR experiments were carried out on a Bruker Ultrashield 300 MHz NMR spectrometer (Bruker Ltd., Milton, ON, Canada) with a 7.05 Tesla magnet. The samples were prepared by dissolving a small amount of the compound into an aliquot of the deuterated solvent under an inert atmosphere. ^1^H and ^13^C{^1^H} spectra were referenced to residual solvent downfield of trimethyl silane. The data were processed using Bruker TOPSIN 1.3.

EPR experiments were performed on a Magnettech GmbH MiniScope 200 X-band EPR spectrometer (Magnettech GmbH, Berlin, Germany) by Juha P. Hurmalainen (University of Jyväskylä, Finland). X-ray measurements were performed at Saint Mary’s University on a Bruker APEXII CCD equipped diffractometer (30 mA, 50 kV, Bruker AXS LLC, Madison, WI, USA) using monochromated Mo Kα radiation (λ = 0.71073 Å) at 125 K. High resolution mass spectrometry was performed on a Bruker microTOF Focus Mass Spectrometer (Bruker Ltd., Milton, ON, Canada) by Xiao Feng at Dalhousie University (Halifax, Nova Scotia, Canada). Gas chromatography for the detection of hydrogen gas was performed by Dr. Zhongmin Dong (Saint Mary’s University) on a custom-built portable gas chromatograph. The GC column (Porapak N 80/100, Alltech Associates Inc.) was linked to a hydrogen sensor (QUBIT Systems Inc.) and to a computer running the Data Logger program (Vernier Software) to collect the data. Melting points were measured using a Mel-Temp melting point apparatus (with a heating rate of ca. 5 °C min^−1^, Barnstead Thermolyne Corp., Dubuque, IA, USA) and are uncorrected. The samples were prepared by filling a capillary tube with a few milligrams of sample material and sealing the tube under an inert atmosphere when the samples were not air stable.

### 3.1. Synthesis of Tetrabutylammonium Thioacetate

#### 3.1.1. In Solution

One mmol of tetrabutylammonium acetate was dissolved in 3 mL of acetonitrile. Two drops of CS_2_ were added, which resulted in the solution turning dark red. This solution was left in a desiccator for the first 24 h and then moved to a glove box where the solvent was allowed to slowly evaporate. After the first 24 h the solution had turned from red to orange. Long, needle crystals that were clear and colorless grew after 3 days. These were removed from the orange gel that remained in the vial. The crystals were identified as tetrabutylammonium thioacetate using FT-IR, ^1^H-NMR and X-ray crystallography. ^1^H-NMR (300 MHz, CD_2_Cl_2_, ppm): 0.97 (t, 12H, ^3^*J*_CH_ = 7.35 Hz, -CH_2_CH_2_CH_2_CH_3_) 1.35 (sep, 8H, ^3^*J*_CH_ = 7.39 Hz, -CH_2_CH_2_CH_2_CH_3_)1.60 (m, 8H, -CH_2_CH_2_CH_2_CH_3_) 2.15 (s, 2H, SOCCH_3_) 3.09 (t, 8H, ^3^*J*_CH_ = 8.44, -CH_2_CH_2_CH_2_CH_3_). IR (KBr, cm^−1^): 2960(vs), 2875(s), 2734(vw), 1710(m), 1678(m), 1590(m), 1553(s), 1493(s), 1468(s), 1385(s) 1341(w), 1270(w), 1227(w), 1156(m), 1031(m), 1110(s), 1031(m), 947(s), 882(w), 804(m), 745(s), 671(m), 617(w), 518(s, br). HRMS calcd. [*m*/*z*]: 339.2028; found [*m*/*z*]: 339.2074.

#### 3.1.2. In the Solid State

One mmol of tetrabutylammonium acetate was placed in a 100 mL Schlenk flask which was sealed under vacuum with a rubber septum. Approximately 5 mL of carbon disulfide was placed in a separate 100 mL Schlenk flask that was evacuated and then refilled with oxygen-free nitrogen gas. A needle was used to remove 5 mL of the gas from the carbon disulfide Schlenk flask, and this was injected into the tetrabutylammonium acetate flask. The solid was allowed to react until the full color change of the solution phase reaction had been observed (colorless to red to orange), then the flask was evacuated again, and the product recovered. ^1^H-NMR (300 MHz, DMSO-*d*_6_, ppm): 0.93 (t, 12H, ^3^*J*_CH_ = 7.35 Hz, -CH_2_CH_2_CH_2_CH_3_), 1.30 (sep, 8H, ^3^*J*_CH_ = 7.39 Hz, -CH_2_CH_2_CH_2_CH_3_) 1.56 (m, 8H, -CH_2_CH_2_CH_2_CH_3_) 1.76 (s, 3H, SOCCH_3_) 3.16 (t, 8H, ^3^*J*_CH_ = 8.44, -CH_2_CH_2_CH_2_CH_3_). IR (gas chamber, NaCl windows, cm^−1^): 2960 (vw), 2360 (w), 2333 (w), 2319 (w), 2192 (m), 2179 (m), 2071 (m), 2052 (w), 1576 (w), 1545 (s, br), 1261 (w), 1017 (w), 885 (w), 799 (w), 670 (w), 621 (w), 464 (s), 448 (s), 419 (s), 409 (s).

### 3.2. Synthesis of bis(triphenylphosphoranylidene)ammonium Formate

In a 250 mL Erlenmeyer flask, 2.5 g of bis(triphenylphosphoranylidene)ammonium chloride ([PNP]Cl) was dissolved in 100 mL of boiling water. In a 1 L Erlenmeyer flask 24.8 g of sodium formate (NaHCOO) was dissolved in 300 mL of boiling water. Both solutions were stirred throughout the reaction. The [PNP]Cl solution was added to the NaCHOO solution in small portions, allowing the solution to clear between each addition. When needed, extra water was added to the solution to clear it, leading to a final solution volume of 800 mL. At this point a small amount of white precipitate could be observed. The flask was removed from the heat and cooled to room temperature before being stored at 5 °C for two hours. The solid was then collected by vacuum filtration. The product was rinsed with three 20 mL portions of diethyl ether. Melting point, ^1^H-NMR and FT-IR were collected on the solid. Mp = 84–87 °C. ^1^H-NMR (300 MHz, CD_3_CN, ppm): 7.56 (m, 30H, Ar), 8.56 (s, 1H, CHO_2_). IR (KBr, cm^−1^): 3658(w), 3052(m), 3022(m), 2958(w), 2809(w), 1610(s), 1586(s), 1482(m), 1436(s), 1370(w), 1345(m), 1310(m, br), 1274(s,br), 1182(m), 1160(w), 1114(s), 1023(w) 997(m), 798(w), 763(m), 749(m), 726(s), 694(s), 616(w), 536(s), 499(s), 457(w), 446(w).

### 3.3. Reaction of bis(triphenylphosphoranylidene)ammonium Formate with SO_2_

#### 3.3.1. In Solution

[PNP][HCO_2_] (0.9 g) of was dissolved in 15 mL of acetonitrile in a 100 mL Schlenk flask. The flask was then evacuated and filled with SO_2_. The solution, which immediately turned a dark royal blue, was allowed to stir overnight before being evacuated again and opened to the atmosphere. Crystals formed in the solution as the solvent evaporated and these were characterized as [PNP][HSO_4_] using FT-IR and ^1^H-NMR. X-ray crystallography confirmed these results, but the structure is not included here and will be published subsequently. ^1^H-NMR (300 MHz, CD_3_CN, ppm): 7.53 (m, 30H, Ar), 8.65 (s, 1H, HSO_4_). IR (NaCl plates, cm^−1^): 3622(w,br), 3541(w), 3202(w), 3003(m), 2944(m), 2293(m), 2253(vs), 1632(w, br), 1441(s, br), 1376(s), 1334(w), 1170(w), 1039(m), 919(m), 750(w), 726(w), 696(w).

#### 3.3.2. In the Solid State

Approximately 0.1 g of [PNP][HCO_2_] was placed in a 50 mL Schlenk flask which was evacuated and then filled with SO_2_. The solid immediately turned dark brown, bubbled, and gave off heat. FT-IR and ^1^H-NMR were used to characterize the solid. FT-IR spectra of the atmosphere above the reaction were taken as the reaction progressed. The same reaction was also repeated in an IR gas chamber. ^1^H-NMR (300 MHz, CD_3_CN, ppm): 7.56 (m, 24H, Ar), 7.71 (m, 6H, Ar). IR (KBr, cm^−1^): 3495(w,br), 3057(m), 3022(m), 1657(w,br), 1587(m), 1482(s), 1439(s), 1251(s, br) 1181(s) 1114(s), 1058(s), 1012(s), 997(s) 832(m), 800(m), 750(s), 724(s), 693(s), 642(m), 593(m), 552(s), 532(s),497(s), 443(w). IR (gas chamber, NaCl window, cm^−1^): 2963 (w), 1529 (w), 1373 (vs), 1357 (vs), 1166 (m), 1137 (m) 799 (w), 623 (w) 454 (s), 432 (s).

### 3.4. Infrared Kinetics Study

In situ infrared spectra were collected using a Mettler Toledo ReactIR 15. All trials were set up using 15 mL of anhydrous acetonitrile and the necessary amounts of tetrabutylammonium acetate and CS_2_. The rate was determined by allowing the reaction to run until there was no longer any change in the height of the peak at 1592 cm^−1^ in the infrared spectrum. This was taken to be the point of complete conversion of the starting material, as CS_2_ was present in excess for each reaction. The percentage of the tetrabutylammonium acetate converted was then calculated the for times between the initial concentration and final endpoint, and rates of reaction determined. The complete set of experiments was then repeated with the tetrabutylammonium acetate present in excess and the quantity of CS_2_ being limited.

### 3.5. Gas Chromatographic Identification of Hydrogen Gas

A solution of [PNP][HCO_2_] in acetonitrile was reacted with SO_2_ gas in a Schlenk flask sealed with a rubber septum. Once the reaction had begun and the solution had turned yellow in colour, a needle was used to extract a sample of gas from the atmosphere above the reaction in the flask. The sample was injected into the custom-built portable gas chromatograph for analysis. In a second reaction, a similar volume of pure hydrogen gas was injected into the same GC. The retention times of both samples were identical. Finally, a sample from the reaction atmosphere and hydrogen gas were combined and run through the GC. This experiment gave one signal with a retention time identical to those observed in the previous runs. The relative volume of the peak was larger than in the individual runs. This was judged to be sufficient proof that hydrogen gas was being produced in the reaction of the formate anion and SO_2_.

### 3.6. EPR Analysis of the Reaction of Na[HCO_2_] and 18-Crown-6 with SO_2_

Sodium formate and an excess of 18-crown-6 were dissolved in 15 mL of acetonitrile in a 100 mL Schlenk flask. The flask was then evacuated and filled with SO_2_. A septum was placed on either end of a flat cell and a needle inserted to allow argon gas to pass through the cell for 10 min. A needle was then used to insert a small portion of the liquid sample. The sample was allowed to run to the bottom before the cell was sealed, after which the spectrum was collected. The spectrum had a single peak at 2.006 g and a half height of 2.9 G.

The solid-state spectrum was collected by mixing together solid sodium chloride, 18-crown-6 and sodium formate. This mixture was exposed to an atmosphere of SO_2_ before evacuating the flask and transferring the solid to an EPR tube under an inert atmosphere. Multiple scans were performed to cancel out the noise and produce a spectrum containing two weak signals at 2.006 g and 2.000 g.

### 3.7. X-ray Crystallographic Analysis of Tetrabutylammonium Thioacetate

The sample was clear and colorless and was obtained in the form of one very large rod-shaped crystal. While attempting to cut this crystal, it broke into a number of smaller, irregularly shaped pieces, one of which was used for analysis. It was attached to the tip of a 300 μm MicroLoop with paratone-N oil. Measurements were made on a Bruker APEXII CCD equipped diffractometer (30 mA, 50 kV) using monochromated Mo Kα radiation (λ = 0.71073 Å) at 125 K [40]. The initial orientation and unit cell were indexed using a least-squares analysis of a random set of reflections collected from three series of 0.5° ω-scans, 10 s per frame and 12 frames per series, that were well distributed in reciprocal space. For data collection, four ω-scan frame series were collected with 0.5° wide scans, 30 s frames and 366 frames per series at varying φ angles (φ = 0°, 90°, 180°, 270°). The crystal to detector distance was set to 6 cm and a complete sphere of data was collected. Cell refinement and data reduction were performed with the Bruker SAINT software, which corrects for beam inhomogeneity, possible crystal decay, Lorentz and polarization effects [41]. A multi-scan absorption correction was applied (SADABS) [42]. The structure was solved using direct methods (SHELXT) [43] and refined using a full-matrix least-squares method on *F*^2^ with SHELXL2014 [44]. The non-hydrogen atoms were refined anisotropically. Hydrogen atoms bonded to carbon were included at geometrically idealized positions and were not refined. The isotropic thermal parameters of the hydrogen atoms were fixed at 1.2 *U*_eq_ of the parent carbon atom, or 1.5 *U*_eq_ for methyl hydrogens. The hydrogens on the terminal methyl group of the anion were observed to be disordered in a near final Fourier map. They were refined using an idealized disordered model where the two sets of methyl hydrogen atoms were rotated from each other by 60° and every hydrogen was given an occupancy of one-half. All of the crystallographic diagrams included in this text were prepared using the program Mercury CSD (Version 3.7) [45]. The cif file CCDC-1524184 has been included in the Supplementary Materials. These data can also be obtained free of charge from the Cambridge Crystallographic Data Centre via www.ccdc.cam.ac.uk/data_request/cif (accessed on 29 November 2021).

## 4. Conclusions

From a matrix of common reactants, we have formed products that suggest that the chemistry of small inorganic ions may have served as a source for important biological molecules in the prebiotic environment. We have performed these reactions under anhydrous conditions, suggesting that they may have occurred at the interface with an aqueous system. It is clear that small molecule–ion interactions must have been key to the origin of many important prebiotic processes and that these reactions have been, until now, overlooked.

## Data Availability

The data presented in this study are available on request from thecorresponding author.

## Supplementary Materials

All data needed to evaluate the conclusions in the paper are present in the paper or in the Supplementary Materials. This includes: Figure S1: Gas chamber infrared spectra from the atmosphere above the reaction of solid tetrabutylammonium acetate and CS_2_ gas. Figure S2: ReactIR monitoring of the reaction between tetrabutylammonium acetate and CS_2_ in acetonitrile solution. Figure S3: Results from the kinetic study of the reaction between tetrabutylammonium acetate and CS_2_. Figure S4: Solid state structure of tetrabutylammonium thioacetate. Figure S5: Crystal packing in the structure of tetrabutylammonium thioacetate. Figure S6: Intermolecular C-H hydrogen bonding in tetrabutylammonium thioacetate drawn from the perspective of one central cation. Figure S7: Intermolecular C-H hydrogen bonding in tetrabutylammonium thioacetate drawn from the perspective of one central anion. Figure S8: Gas chamber infrared spectra from the atmosphere above the reaction of solid bis(triphenylphosphoranylidene)ammonium formate and SO_2_ gas. Figure S9: ReactIR monitoring of the loss of SO_2_ (top) and detection of the formation of CO_2_ (bottom) for the reaction of bis(triphenylphosphoranylidene)ammonium formate and SO_2_ in acetonitrile solution. Figure S10: Solution EPR from the reaction of sodium formate,18-crown-6, and sulfur dioxide in acetonitrile. Table S1: Crystal data and structure refinement details for tetrabutylammonium thioacetate. Table S2. C-H…X (X = S or O) hydrogen bonds to the sum of the van der Waals radii + 0.20 Å for tetrabutylammonium acetate [Å and °]. The cif file CCDC-1524184 has been deposited as a Supplementary Material or it can be obtained free of charge from the Cambridge Crystallographic Data Centre via www.ccdc.cam.ac.uk/data_request/cif (accessed on 29 November 2021).

## Sample Availability

Samples of the compounds are not available from the authors.
