# Supplementary material for: Simple Ion–Gas Mixtures as a Source of Key Molecules Relevant to Prebiotic Chemistry
Source: Molecules. 2021 Dec 6;26(23):7394. doi: 10.3390/molecules26237394 (PMC8659102; doi:10.3390/molecules26237394)
Supplement: Supplementary file 1 [file molecules-26-07394-s001.zip › molecules-1441138-supplementary.pdf]

Supporting Information

for

Simple ion – gas mixtures as a source of key molecules relevant to prebiotic chemistry

Authors:

Samuel Paula, Liam S. Goulding, Katherine N. Robertson and Jason A. C. Clyburne*

Affiliations:

Department of Chemistry, Saint Mary's University, Halifax, Nova Scotia, B3H 3C3, Canada.

Tel: 01 902 420 5827.

*Correspondence to: jason.clyburne@smu.ca;

This manuscript is dedicated to Professor Alan Cowley.

List of Figures in the Supporting Information

Figure S1: Gas chamber infrared spectra from the atmosphere above the reaction of solid tetrabutylammonium acetate and CS_2 gas.

Figure S2: ReactIR monitoring of the reaction between tetrabutylammonium acetate and CS_2 in acetonitrile solution.

Figure S3: Results from the kinetic study of the reaction between tetrabutylammonium acetate and CS_2.

Figure S4: Solid state structure of tetrabutylammonium thioacetate.

Figure S5: Crystal packing in the structure of tetrabutylammonium thioacetate.

Figure S6: Intermolecular C-H hydrogen bonding in tetrabutylammonium thioacetate drawn from the perspective of one central cation.

Figure S7: Intermolecular C-H hydrogen bonding in tetrabutylammonium thioacetate drawn from the perspective of one central anion.

Figure S8: Gas chamber infrared spectra from the atmosphere above the reaction of solid bis(triphenylphosphoranylidene)ammonium formate and SO_2 gas.

Figure S9: ReactIR monitoring of the loss of SO_2 (top) and detection of the formation of CO_2 (bottom) for the reaction of bis(triphenylphosphoranylidene)ammonium formate and SO_2 in acetonitrile solution.

Figure S10: Solution EPR from the reaction of sodium formate,18-crown-6 and sulfur dioxide in acetonitrile.

List of Tables in the Supporting Information

Table S1: Crystal data and structure refinement details for tetrabutylammonium thioacetate.

Table S2. C-H…X (X = S or O) hydrogen bonds to the sum of the van der Waals radii + 0.20 Å for tetrabutylammonium acetate [Å and °].

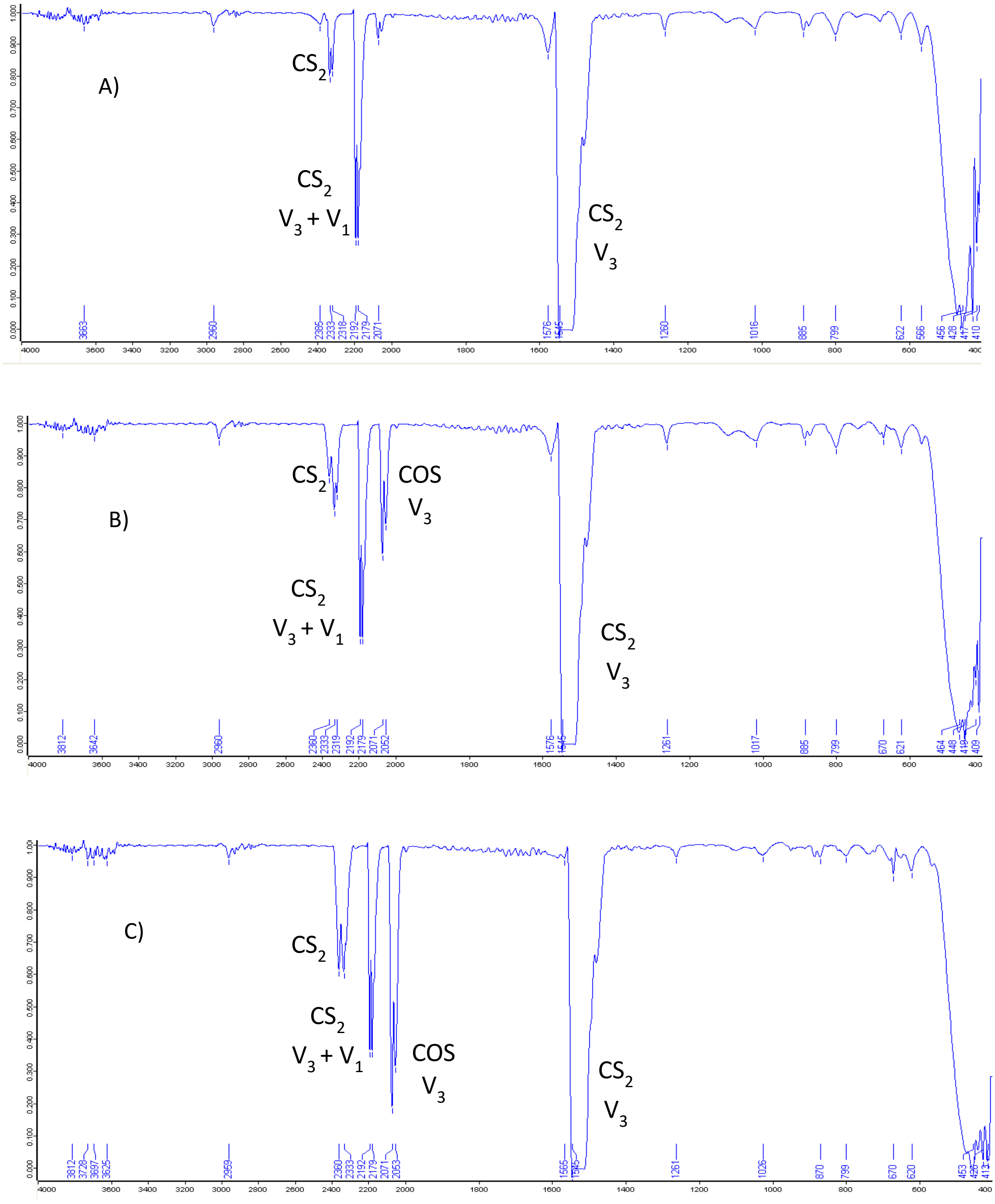

Figure S1: Gas chamber IR spectra from the atmosphere above the reaction of solid tetrabutylammonium acetate and CS_2 gas at A) 2 min B) 2 h and C) 20 h. The series shows a progressive increase in the amplitudes of the peaks at 2071 and 2053 cm^{-1} which were assigned to the ν_3 stretch of COS. This, along with the relative decrease in the intensity of the peaks assigned to CS_2, indicates a reaction between tetrabutylammonium acetate and CS_2 that has an overall exchange mechanism.

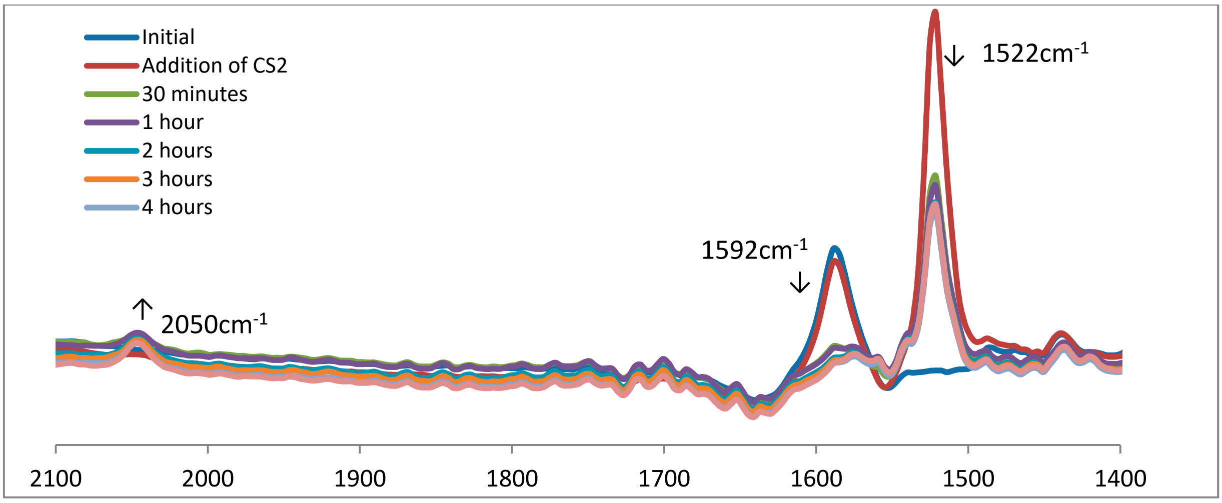

Figure S2: ReactIR monitoring of the reaction between tetrabutylammonium acetate and CS_2 in acetonitrile solution. The changes in the IR spectra show the loss of acetate (1592 cm^{-1}) and CS_2 (1522 cm^{-1}) along with the production of COS (2050 cm^{-1}).

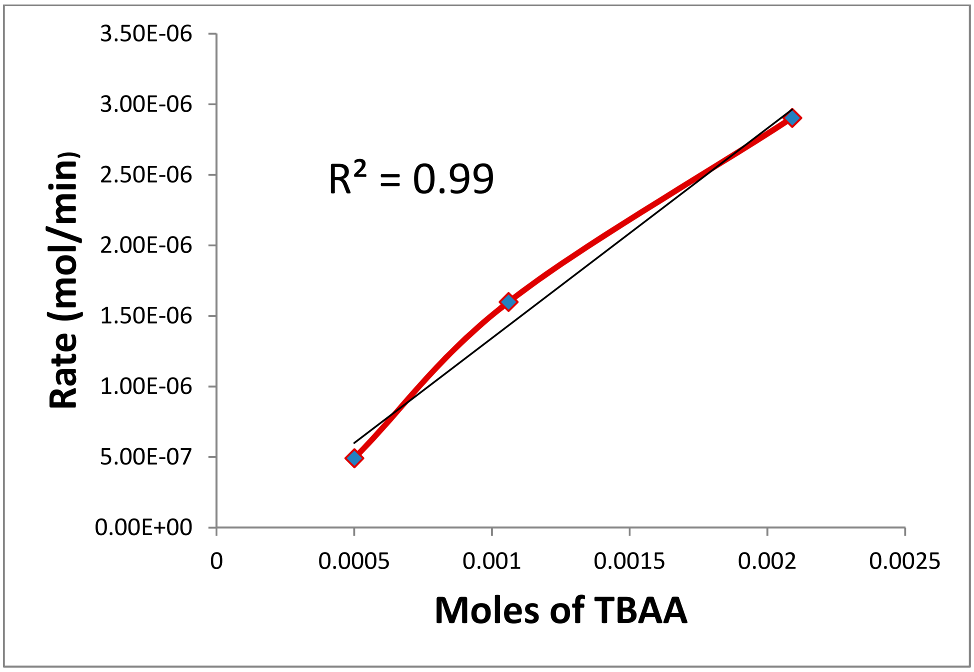

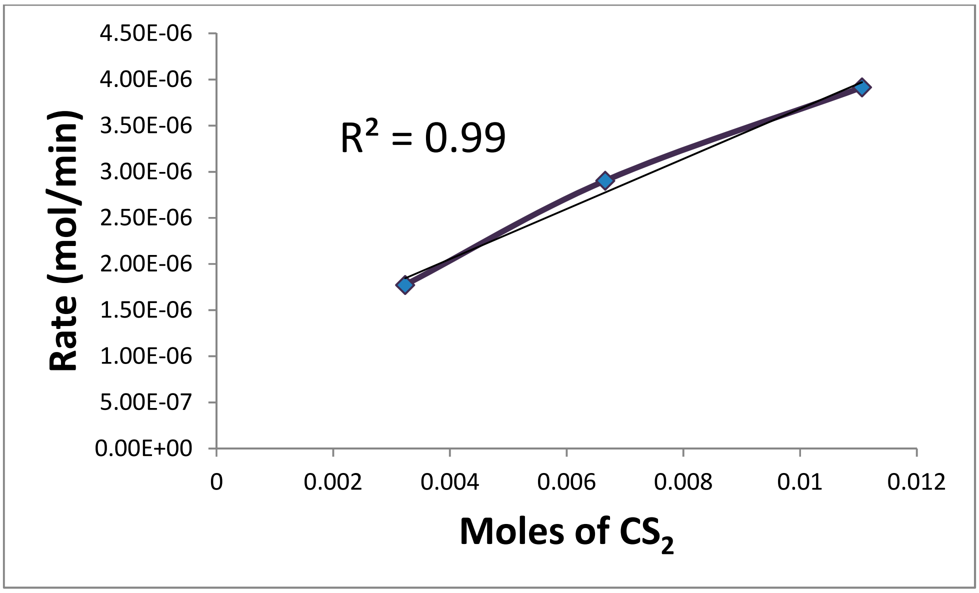

Figure S3: Results from the kinetic study of the reaction between tetrabutylammonium acetate and CS_2, (A) rate of reaction based on moles of tetrabutylammonium acetate (top) and (B) rate of reaction based on moles of CS_2 (bottom).

Table S1: Crystal data and structure refinement details for tetrabutylammonium thioacetate

Empirical formula	$C_{18}H_{39}NOS$	
Formula weight	317.56	
Temperature	125(2) K	
Wavelength	0.71073 Å	
Crystal system	Monoclinic	
Space group	$P2_1/n$	
Unit cell dimensions	a = 9.4186(6) Å	α= 90°
	b = 14.1149(10) Å	β= 103.3250(10)°
	c = 16.0679(11) Å	γ = 90°
Volume	2078.6(2) $Å^3$	
Z	4	
Density (calculated)	1.015 Mg/m^3	
Absorption coefficient	0.157 mm^{-1}	
F(000)	712	
Crystal size	0.220 x 0.180 x 0.120 mm^3	
Theta range for data collection	2.302 to 28.889°	
Index ranges	-12<=h<=12, -18<=k<=18, -21<=l<=21	
Reflections collected	24768	
Independent reflections	5123 [R(int) = 0.0313]	
Completeness to theta = 25.242°	99.9 %	
Refinement method	Full-matrix least-squares on F^2	
Data / restraints / parameters	5123 / 0 / 194	
Goodness-of-fit on F^2	1.016	
Final R indices [I>2sigma(I)]	R1 = 0.0389, wR2 = 0.0917	
R indices (all data)	R1 = 0.0577, wR2 = 0.1010	
Extinction coefficient	n/a	
Largest diff. peak and hole	0.317 and -0.197 e.$Å^{-3}$	

Table S2. C-H…X (X = S or O) hydrogen bonds to the sum of the van der Waals radii + 0.20 Å for tetrabutylammonium acetate [Å and °]

D-H...A	d(D-H)	d(H...A)	d(D...A)	<(DHA)
C(1)-H(1A)...S(1)#1	0.99	2.97	3.8894(12)	154.8
C(1)-H(1A)...O(1)#1	0.99	2.87	3.7651(16)	151.4
C(1)-H(1B)...O(1)	0.99	2.36	3.3317(15)	167.4
C(3)-H(3A)...S(1)#1	0.99	3.16	4.0291(15)	146.9
C(4)-H(4B)...O(1)#3	0.98	2.84	3.7513(18)	155.2
C(5)-H(5B)...S(1)#2	0.99	2.79	3.7518(12)	164.0
C(6)-H(6A)...S(1)#1	0.99	2.90	3.9044(13)	157.8
C(9)-H(9A)...S(1)	0.99	2.98	3.9379(13)	162.7
C(9)-H(9B)...O(1)#1	0.99	2.54	3.4804(15)	159.3
C(11)-H(11A)...S(1)	0.99	3.12	4.0406(15)	154.8
C(14)-H(14A)...S(1)	0.99	3.15	3.9852(13)	143.0
C(14)-H(14B)...O(1)	0.99	2.84	3.4976(16)	124.9

Symmetry transformations used to generate equivalent atoms:
#1 -x+1,-y+1,-z+1   #2 -x+1/2,y+1/2,-z+1/2   #3 -x+2,-y+1,-z+1

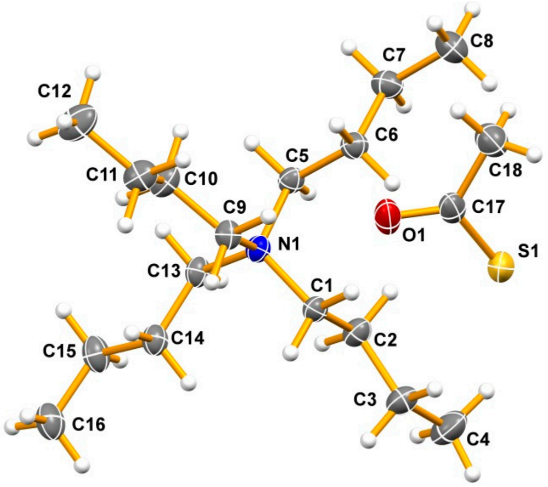

Figure S4: Solid state structure of tetrabutylammonium thioacetate with thermal ellipsoids drawn at the 50% probability level.

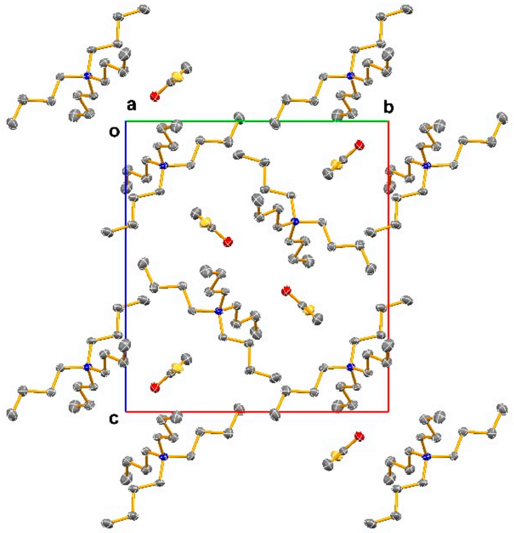

Figure S5: Crystal packing viewed down the *X*-axis in the structure of tetrabutylammonium thioacetate. Thermal ellipsoids have been drawn at the 50% probability level and hydrogen atoms have been omitted for clarity.

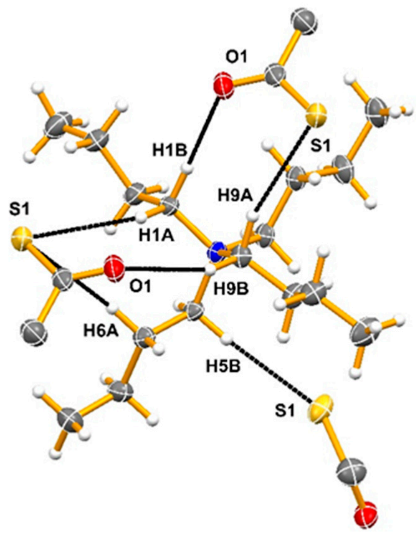

Figure S6: Short intermolecular C-H hydrogen bonding (dotted lines) to the sum of the van der Waals radii in tetrabutylammonium thioacetate (Table S2) drawn from the perspective of one central cation. Thermal ellipsoids have been drawn with 50% probability. Only the atoms involved in the interactions have been labelled.

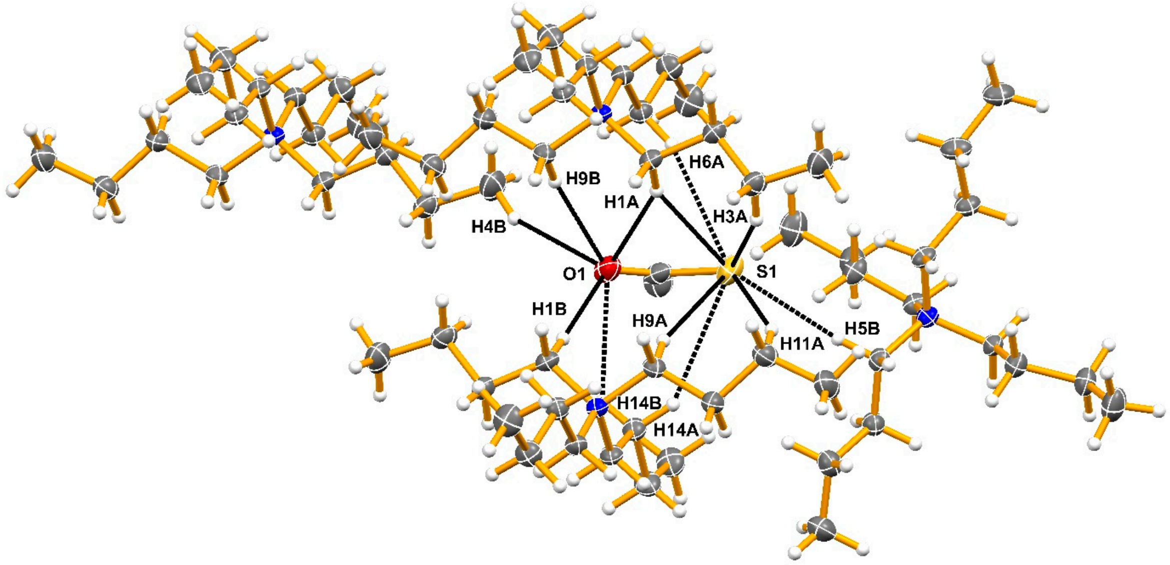

Figure S7: Intermolecular C-H hydrogen bonding (dotted lines) in tetrabutylammonium thioacetate (Table S2) drawn from the perspective of one central anion. Thermal ellipsoids have been drawn with 50% probability. Only the atoms involved in the interactions have been labelled.

An investigation of the intermolecular contacts proved to be revealing. To the limit of the sum of the van der Waals radii of the interacting atoms, a total of 4 C-H···S and 2 C-H···O contacts were identified (Figure S6 and Table S2). There are more close contacts from the cation to the larger, softer sulfur atom in the anion than to the smaller and harder oxygen atom. Each cation interacts with three different anions. In the cation the C-H groups closest to the central nitrogen are the ones most likely to form the closest contacts. Interestingly, H1A(S1) and H9B(O1) interact with one anion, while on the opposite side H1B(O1) and H9A(S1) interact with a second anion, accounting for 4 of the 6 closest contacts in the structure. H5B(S1) which interacts with the third anion is also on a carbon atom directly adjacent to the central nitrogen center. These hydrogen atoms must have the largest induced positive dipoles. Unlike the other branches, the hydrogen atoms on C13 of the fourth carbon chain do not participate in any of the short contacts.

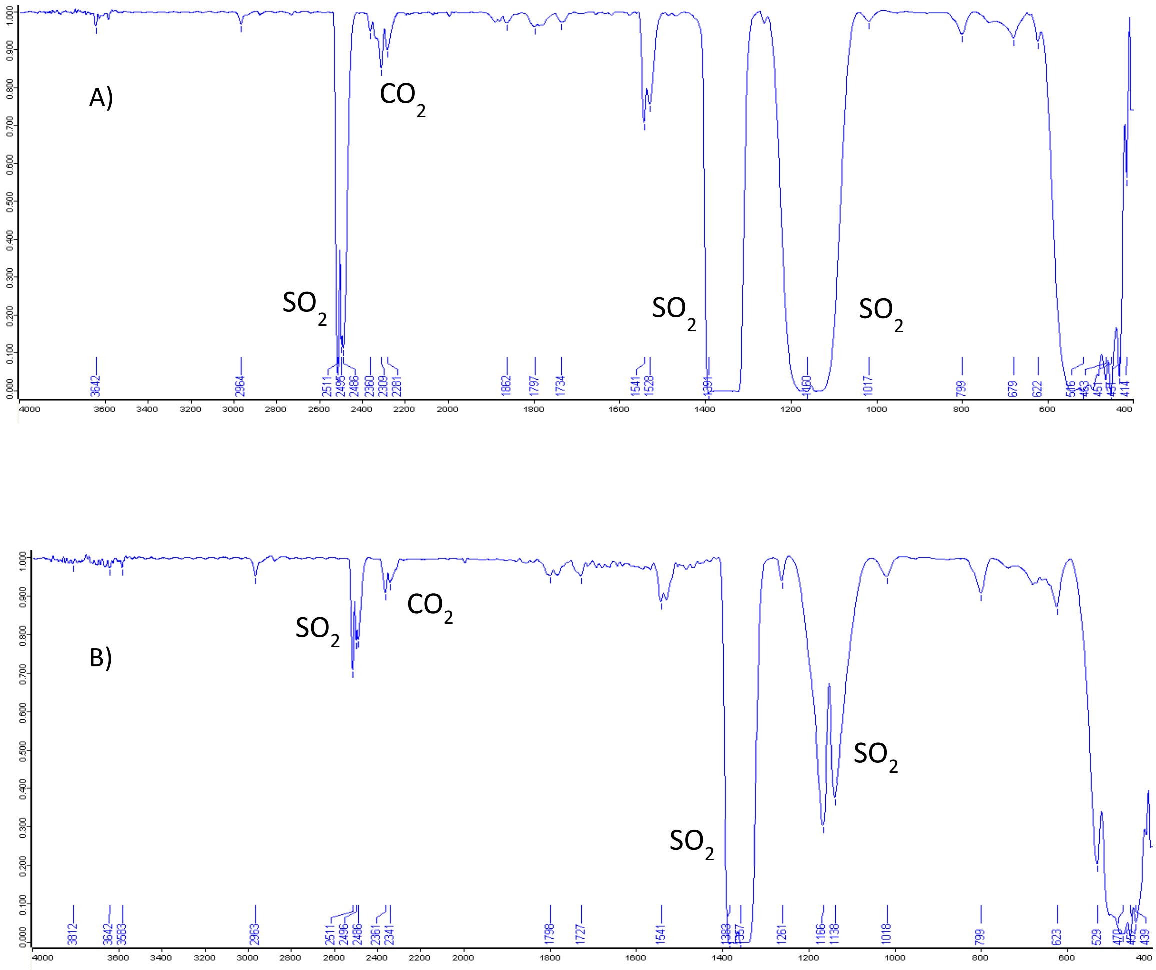

Figure S8: Gas chamber infrared spectra from the atmosphere above the reaction of solid bis(triphenylphosphoranylidene)ammonium formate and SO_2 gas, at A) 2 min and B) 30 min.

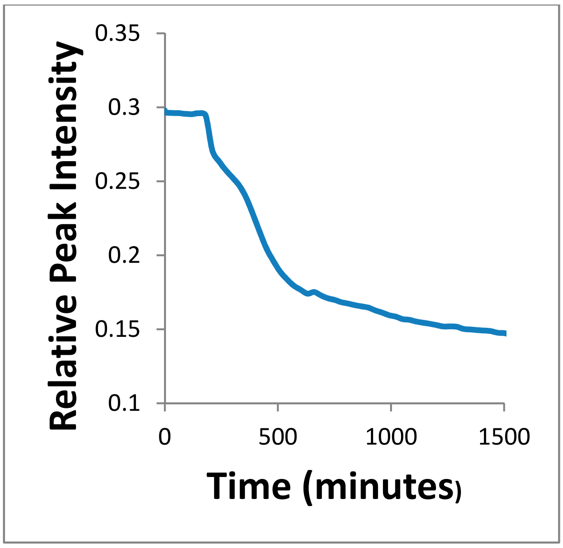

Peak at 1637 cm^{-1}

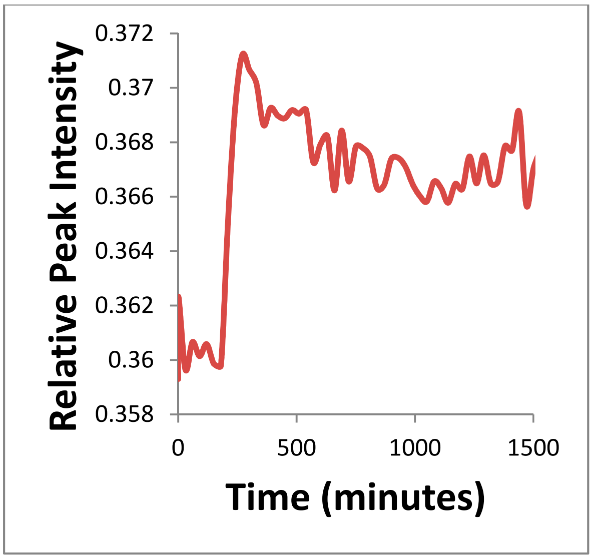

Peak at 2355 cm^{-1}

Figure S9: ReactIR monitoring of the loss of SO_2 (top) and detection of the formation of CO_2 (bottom) for the reaction of bis(triphenylphosphoranylidene)ammonium formate and SO_2 in acetonitrile solution.

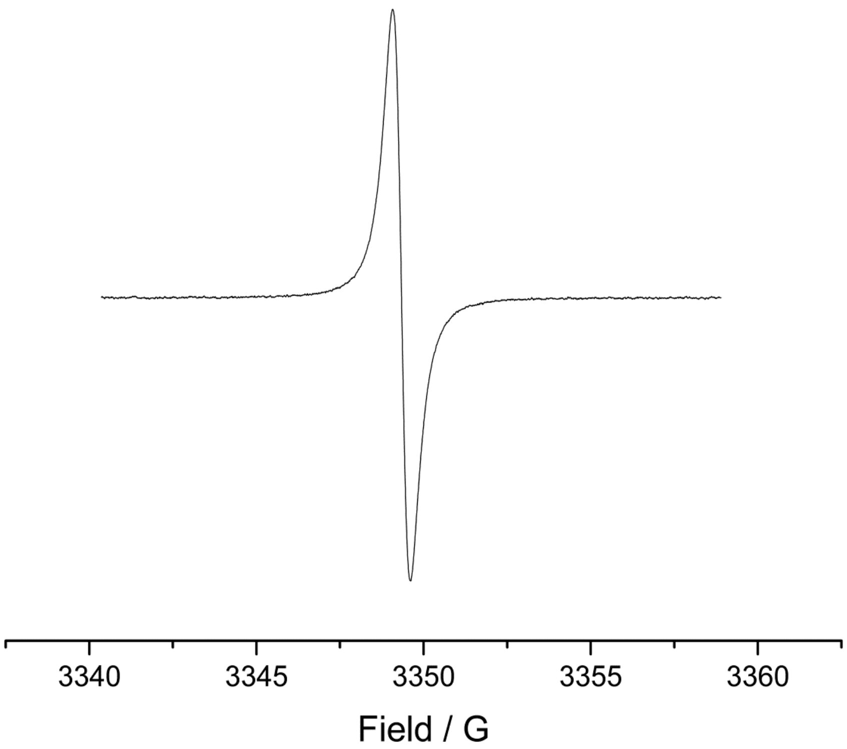

Figure S10: Solution EPR spectrum from the reaction of sulfur dioxide, sodium formate and 18-crown-6 in acetonitrile solution.

An electron paramagnetic resonance (EPR) spectrum was run on the solution phase reaction using sulfur dioxide, sodium formate and 18-crown-6. The single peak observed is in accordance with the reported EPR spectrum of the $SO_2^{\cdot -}$ radical. A much weaker peak with the same shift was observed when a mixture of the solid reagents in NaCl was exposed to SO_2.
